# Supplementary material for: Acoustophoretic system for seed separation on conveyor belts
Source: Nat Commun. 2025 Jul 29;16:6975. doi: 10.1038/s41467-025-62006-3 (PMC12307790; doi:10.1038/s41467-025-62006-3)
Supplement: Supplementary file 1 — Supplementary Information [file 41467_2025_62006_MOESM1_ESM.pdf]

Supplementary Information for Acoustophoretic System for Seed Separation on Conveyor Belts

James Hardwick[1,2], Zak Morgan[1,2] and Ryuji Hirayama[1,2]
[1]Department of Computer Science, University College London, UK, [2]Acoustofab Ltd, London, UK

Supplementary Note 1. COMPARISON WITH TRADITIONAL APPROACHES

The primary alternatives to our system for object ejection on a conveyor belt include pneumatic air jets and mechanical gravity sorting systems. While air-jet sorters provide the most appropriate comparison to our acoustophoretic system due to their similar functional role in performing targeted ejection from monolayer object streams, is also valid to include a comparison to gravity sorters. This is because although they are based on different physical principles, gravity sorters represent a key point of reference for evaluating separation performance across the industry, and including them helps situate our system within the broader landscape of existing technologies.

We compare the performance and efficiency of our acoustophoretic separation system with these conventional technologies, as summarized numerically in Supplementary Table 1. For metrics relevant only to air jet and acoustophoretic systems, gravity separators are excluded from comparison.

Table 1: Comparison of separator parameters.

Parameter	Gravity Separators	Air Jet Separators	Acoustophoretic Separator
Belt Width	∼1–4 m	∼1–3 m	Any
Power Usage	∼1–6.3 kW/m	∼1–2.7 kW/m	0.4 kW/m
Max Ej. Force	-	∼5 N	0.5 N
Ejection Time	-	∼10/sec	50-100/sec
Noise Level	∼72–88 dB	∼70–85 dB	∼55 dB

Note: Gravity separator information was compiled from product data sheets of the Cimbria GA Series, Skiold Damas Zeta, Petkus Gravity Tables, Oliver Manufacturing Voyager Gravity Separator, SensoVision VizSort, and ZBV-SKS Gravity Sorter. Air jet separator information was sourced from data sheets of the Cimbria SEA CX Color Sorter, Bühler Sortex Series, TOMRA 3C Optical Sorter, and Optimum Sorting VENTUS.

Belt Width and Flexibility: Our acoustophoretic ejection system offers design flexibility due to its ability to eject objects at a distance. Unlike pneumatic air jets, which must be positioned within ∼1 cm or less of the target [1], the acoustophoretic system can operate effectively from up to 40 cm away, still generating pressures of 4000 Pa (see Fig.2b). This means that they can be positioned in many locations relative to the rest of the sorting setup and still perform their separating work.

Energy Consumption: Secondly, our system significantly reduces energy consumption compared to traditional mechanical and pneumatic sorting systems. We measure power consumption in kilowatts per meter of belt (kW/m) to provide a fair basis for comparing energy efficiency across different sorting technologies, as our modular system can be tiled to any width. Gravity sorters consume 1–6.3 kW/m due to the high energy demands of their mechanical components, such as vibrating screens, as well as the power required to move materials against gravity. Similarly, pneumatic air jet separators require around 1–2.7 kW/m, primarily to maintain a constant supply of compressed air, which constitutes a substantial part of their operational costs. In contrast, our phased array transducer system averages only 0.4 kW/m of belt. The lower energy requirements of our system not only enhance its cost-effectiveness, especially for large-scale operations, but also underscore its operational efficiency compared to gravity sorters and pneumatic air jets, where maintaining energy-intensive processes can significantly escalate overall costs.

Cost: Our approach is designed to be cost-effective in both initial setup, running costs and ongoing maintenance. Gravity sorters typically range from $10,000 to over $30,000/unit, depending on their belt width, capacity

and complexity. In comparison, pneumatic air jet sorters generally start at around $20,000 and can exceed $200,000/unit for larger or more advanced systems[1]. However, the majority of this cost lies in the vision systems, with the separation component representing a smaller portion of the overall expense. A typical cost for an air-jet nozzle is around $100, and 50–100 nozzles are typically required to cover the full width of a conveyor belt. With an additional compressor cost of ∼$1,000, the total cost of an air-jet separation system ranges from $6k to $11k. While the exact market price of our ejection system has not yet been finalized, we are confident that it will be competitive and likely lower than these because the main components of the acoustophoretic ejection system are cheap ultrasound transducers, which are widely available as car parking sensors. Our system's contactless actuation approach reduces wear-and-tear on components compared to mechanical methods like gravity sorting, which can require frequent maintenance and replacements of parts. Unlike air jet separators, our system avoids dust clogs, a common issue that increases maintenance need for pneumatic systems. This is further reduced by its minimal mechanical components compared to pneumatic systems. The lower upfront costs, energy savings, and independence from compressed air, along with reduced maintenance expenses, contribute to a significantly lower total cost of ownership, establishing our acoustophoretic separator as a cost-effective alternative to both gravity sorters and pneumatic air jets.

Response Time and Ejection: The maximum ejection forces of pneumatic air jets and acoustophoretic separation systems vary significantly, affecting their sorting effectiveness. Gravity sorters separate using mechanical movement rather than targeted ejection forces and as such, making direct comparisons with pneumatic and acoustophoretic systems is not meaningful for these parameters. Pneumatic air jets can generate ejection forces of up to 5 N, while our acoustophoretic system produces a lower force of approximately 0.5 N. However, this reduced force is sufficient for sorting small, lightweight objects (such as seeds), which require forces much lower than this to alter their trajectories. Conventional optical sorting machines achieve ejection rates of up to 10 objects per second[2], with pneumatic air jet nozzles covering a few cm^2 each and a full array spanning several hundred cm^2. This rough estimate provides a general idea of the ejection times currently achievable, though pneumatic systems can vary depending on material, nozzle configuration, and air pressure. In contrast, our acoustophoretic separation system achieves 50–100 ejections per second with a 10–20 ms ejection time over a phased array board spanning several hundred cm^2. While variations in nozzle size and pitch among air jet separators, as well as differences in acoustophoretic separator area, complicate direct comparisons, rough estimates suggest that the ejection capabilities of acoustophoretic systems are comparable to those of pneumatic systems, indicating their potential for high-throughput sorting.

Accuracy: Comparing the accuracy of gravity, air jet, and acoustophoretic separation systems reveals notable differences in error rates, precision, and spatial flexibility. Gravity sorters, which separate by weight, often misclassify seeds with similar densities, leading to both loss of viable seeds and retention of unwanted material. Air jets improve precision by directing compressed air at detected seeds, but fixed jet positions limit them to specific points in front of each nozzle. This setup not only creates gaps in operational coverage but also risks ejecting neighboring seeds along with targets. In contrast, acoustophoretic systems achieve high accuracy by directing acoustic pressure precisely onto a target seed, regardless of its position within the device's operational volume, thanks to the redirectability of their interference-based pressure distribution. This approach minimizes impact on adjacent particles. Acoustic beams, as narrow as 15 mm (see Supplementary Fig. 2a), allow precise targeting of individual seeds, providing flexible, continuous coverage well-suited to delicate, accurate sorting tasks.

Noise: Finally, our acoustophoretic sorting system operates quietly, primarily generating sound from the modulation of the 40 kHz ultrasound signal at approximately 55 dB, comparable to moderate conversation. While this noise level may increase slightly with higher ejection rates, it remains significantly lower than that of pneumatic air jets, which can reach 70 to 85 dB. Although gravity sorters are generally quiet, they produce noise mainly from mechanical movement, typically ranging from 50 to 70 dB. Overall, the acoustophoretic system offers a significant advantage in noise reduction, making it an ideal choice for environments sensitive to sound pollution.

---

[1] Note: These prices were estimated based on listed market prices seen on online re-salers

[2] See: SensoVision VizSort.

Supplementary Note 2. PHASED ARRAY TRANSDUCER SYSTEM

As described in Section 2 of the main text, the core of our system is ultrasonic phased array transducers (PATs), as shown in Supplementary Fig. 1a. Our PAT board consists of 16 × 16 ultrasound transducers, each of which has a diameter of 10 mm and emits an ultrasound wave at a frequency of 40 kHz when driven by a 40-kHz square wave signal (see Supplementary Fig. 1b). The phase of each transducer ϕ_t can be controlled by delay this square wave signal. For example, delaying the signal for 12.5 μs results in the phase delay of π radians, given that the period of the 40-kHz wave is 25 μs. The amplitude of each transducer a_t, ranging from 0 to 1, can be adjusted by changing the duty cycle (%) of the square wave signal [2] as follows:

$$a_t = \sqrt{\sin^2\left(\frac{duty}{100}\pi\right)}. \tag{1}$$

Thus, a 50% duty cycle gives the maximum amplitude output, while a 0% duty cycle provides no sound wave.

In our system, the FPGA board mounted on the PAT board generates such square waves based on data received via the USB bridge (see Supplementary Fig. 1c), providing 128 levels of phase resolution and 64 levels of amplitude resolution. Due to the limited number of input/output pins available on the FPGA board, the 256 transducers are divided into 32 groups, each consisting of eight transducers. The FPGA multiplexes eight square-wave signals into a single signal for each group, which is then sent to 32 shift registers. The shift registers de-multiplex the signals, restoring the original eight square waves for each group. These de-multiplexed signals are subsequently amplified by MOSFETs to 20 Vpp to drive the transducers.

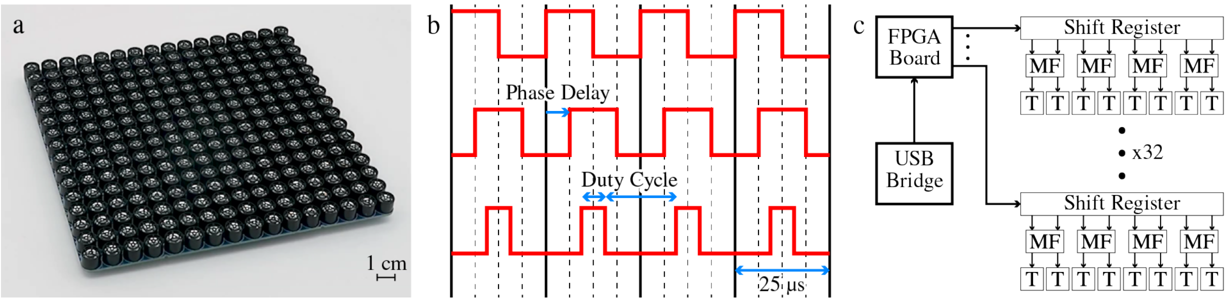

Figure 1: System diagram of the phased array of transducers (PAT). **(a)** Overview of the PAT board. **(b)** Square-wave signals to control the phase and amplitude of each transducer. **(c)** Block diagram of the PAT board. MF: MOSFET, T: Transducer.

Supplementary Note 3. FIELD GENERATED BY THE PHASED ARRAY TRANSDUCERS

First, consider the sound field generated at a point $\mathbf{x_p}$ by a single transducer located at $\mathbf{x_t}$, activated with a specific amplitude a_t and phase ϕ_t (see Supplementary Fig. 2). Due to the directivity of the transducer and sound attenuation, the amplitude pressure at the point a_p can be represented using a piston model as follows:

$$a_p = \frac{2J_1(kr\sin(\theta_{p,t}))}{kr\sin(\theta_{p,t})}\frac{P_{ref}}{d_{p,t}}a_t. \tag{2}$$

Here, k is the wave number; r is the transducer's radius; P_{ref} is the transducer's reference pressure at 1-m distance when amplitude is maximum (i.e., $a_t = 1$); $d_{p,t}$ is the Euclidean distance between the transducer at $\mathbf{x_t}$ and the point $\mathbf{x_p}$ (i.e., $|\mathbf{x_p} - \mathbf{x_t}|$); $\theta_{p,t}$ is the angle between the transducer's normal and the point $\mathbf{x_p}$; and $J_1()$ is the Bessel function of the first kind. The phase at the point ϕ_p is determined by the propagation distance $d_{p,t}$ and can be represented as:

$$\phi_p = kd_{p,t} + \phi_t. \tag{3}$$

Using complex notation ($\zeta_p = a_p e^{i\phi_p}$ and $\tau_t = a_t e^{i\phi_t}$), this model can be expressed as:

$$\zeta_p = \frac{2J_1(kr\sin(\theta_{p,t}))}{kr\sin(\theta_{p,t})}\frac{P_{ref}}{d_{p,t}}e^{ikd_{p,t}}\tau_t = F_{p,t}\tau_t. \tag{4}$$

Based on the principle of wave superposition, the sound field generated at a point $\mathbf{x_p}$ from all T transducers of the array can be expressed as a summation of their contributions:

$$\zeta_p = \sum_{t=1}^{T} F_{p,t}\tau_t. \tag{5}$$

Using vector representations ($\boldsymbol{\zeta} = \{\zeta_1, \zeta_2, ..., \zeta_P\}$, $\boldsymbol{\tau} = \{\tau_1, \tau_2, ..., \tau_T\}$), the sound field at P different points generated by the array of T transducers can be represented as a simple linear equation system:

$$\boldsymbol{\zeta} = \begin{bmatrix} F_{1,1} & \cdots & F_{1,T} \\ \vdots & \ldots & \vdots \\ F_{P,1} & \cdots & F_{P,T} \end{bmatrix} \boldsymbol{\tau} = \mathbf{F}\boldsymbol{\tau}. \tag{6}$$

This linear equation, which uses the forward transmission matrix $\mathbf{F}$, is useful when creating multiple focal points simultaneously, as described in the following section.

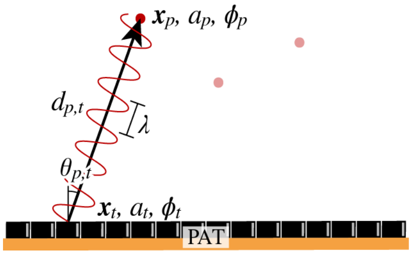

Figure 2: Illustration of the sound field generated by the phased array transducers.

Supplementary Note 4. FOCUSING ALGORITHM

As described in Section 4 of the main text, the phase of each transducer ϕ_t, required to create an amplitude focal point at $\mathbf{x_p}$, can be determined as:

$$\phi_t = -kd_{p,t} + \phi'_p. \tag{7}$$

Here, the wave number k is related to the wavelength λ as $k = 2\pi/\lambda$, and $d_{p,t}$ represents the distance between the transducer at $\mathbf{x_t}$ and the focal point at $\mathbf{x_p}$. Due to the differences in the propagation distances $d_{p,t}$, sound waves emitted by the transducers simultaneously reach the target point at slightly different times (i.e., different number of waves inside the propagation distance). This timing difference introduces phase differences at the target point between the contributions from each transducer that emits the ultrasound wave simultaneously. Eq. 7 accounts for these timing differences, ensuring constructive interference of the waves at the desired target position. This ensures that the waves emitted by all the transducers reach the desired target position simultaneously, resulting in a high-pressure acoustic focal point with the target phase ϕ'_p. This can be confirmed by substituting Eq. 7 into Eq. 3, which yields $\phi_p = \phi'_p$. When all the transducer amplitudes a_t are set to its maximum (i.e., $a_t = 1$), the PAT generates the maximum focal point amplitude a_p for that target location $\mathbf{x_p}$. The focal point amplitude a_p can be easily controlled by adjusting a_t, for example, the PAT generates half of the maximum pressure for that point when setting $a_t = 0.5$ for all the transducers.

To create multiple focal points, the most straightforward method involves using a backward transmission matrix $\mathbf{B}$, which is the conjugate transpose matrix of $\mathbf{F}$. Given the target sound field at a set of points $\boldsymbol{\zeta}$, the activation

of the transducers $\boldsymbol{\tau}$ can be determined in two steps. First, the target sound field $\boldsymbol{\zeta}$ is back-propagated to the transducers as:

$$\boldsymbol{\tau} = \mathbf{B}\boldsymbol{\zeta}. \tag{8}$$

Then, the amplitude information is discarded to get maximum pressure as:

$$\tau_t = \frac{\tau_t}{|\tau_t|}, \text{for } t = 1, 2, ..., T. \tag{9}$$

Here, $|\tau_t|$ is a non-zero value.

While this simple method allows for the simultaneous creation of multiple focal points, it risks introducing destructive interference between the points, leading to unbalanced focal point strengths. This occurs primarily because the target point phases ϕ'_p are often arbitrarily set (e.g., all set to 0 rad). To avoid such destructive interferences, phase retrieval algorithms are commonly used to get optimal target point phases. The most popular methods are based on the Gerchberg-Saxton (GS) algorithm from the optics community [25]. These algorithms heuristically compute the target point phases ϕ'_p that optimize field reconstruction by iteratively performing the forward- and backward-propagation processes (Eq. 6 and Eq. 8) while constraining the amplitudes of both the transducers and the focal points.

Supplementary Note 5. OBJECT PRE-ARRANGEMENT

In addition to its object-ejection capabilities, the PAT board can be used for other sorting applications, such as pre-arrangement of objects on conveyor belts. In this application, the PAT board is positioned above the conveyor belt, as shown in Supplementary Fig. 3a. Using the same algorithm (Eq. 7, the PAT board generates a focal point at the location of a seed. Due to the presence of the conveyor belt in the direction of sound propagation, the seed cannot be ejected. Instead, the focal point on the belt's flat surface creates a levitation trap (i.e., standing-wave pattern [4]), suspending the seed at a height of $\lambda/4$ (=$\sim$ 2 mm) above the belt. By moving this levitation trap, the seed can be manipulated along the surface while hovering.

As described in Section 3 of the main text, this hovering capability is useful for pre-arranging seeds on conveyor belts. Supplementary Figs. 3b and 3c show preliminary results obtained using a PAT board with a flat reflector (instead of using a conveyor belt). Nine pepper seeds were randomly positioned on the surface. Our imaging system detected the seeds' positions, and the PAT board then moves each seed to the nearest point on a 3 × 3 grid, one by one. This approach has a potential to improve the efficiency of the later part of the sorting process (i.e., ejection).

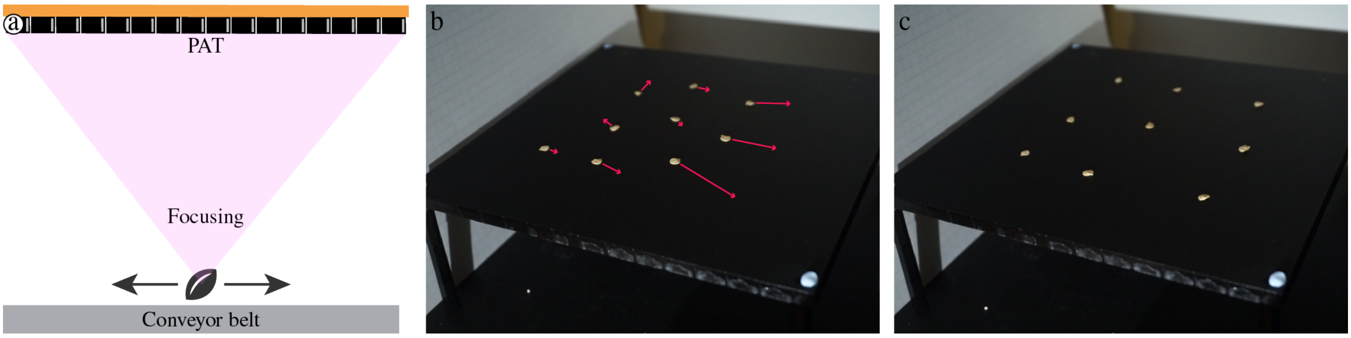

Figure 3: Preliminary results of the pre-arrangement application. **(a)** Setup overview. Note that the belt is moving in the direction of the figure's depth. **(b)** Nine pepper seeds randomly positioned. **(c)** Sorted seeds on a 3 × 3 grid.

Supplementary Note 6. 3D INSPECTION, COATING, PELLETING, AND PRIMING

For applications like 3D inspection, coating, pelleting, and priming, it is essential to lift seeds from a conveyor belt. While the setup like Supplementary Fig. 3a is suitable for hovering seeds along the surface, it is not capable to lift seeds up from the conveyor belt due to the influence of strong sound scattering near the surface [3]. The setup with two PAT boards on the sides of the belt as shown in Supplementary Fig. 4 is more suitable for these type of applications. Here, simply focusing on the seed location is not enough to lift the seed up from the surface due to the sound scattering from the surface. To account for such scattering, an algorithm using a reformulation of the Boundary Element Method (BEM) was proposed [3]. This algorithm first models the contributions of each transducer to target points using the reformulated BEM and then use this model to solve for the transducers' phases ϕ_t to minimizing the simplified Gor'kov potential U [5] at the desired trapping positions. We use the same algorithm to trap and manipulate a seed with our setup.

To demonstrate the ability of this setup to lift a seed up from the surface, we conducted a preliminary test using a pepper seed. As shown in Supplementary Fig. 4b and 4c, our setup could successfully lift the pepper seed up from the surface. While we used an acrylic sheet as a reflector in this experiment, the same result can be obtained by replacing it with a conveyor belt. This preliminary result shows the potential of PATs to be used for more sophisticated applications like 3D inspection, coating, pelleting, and priming of seeds in mid-air.

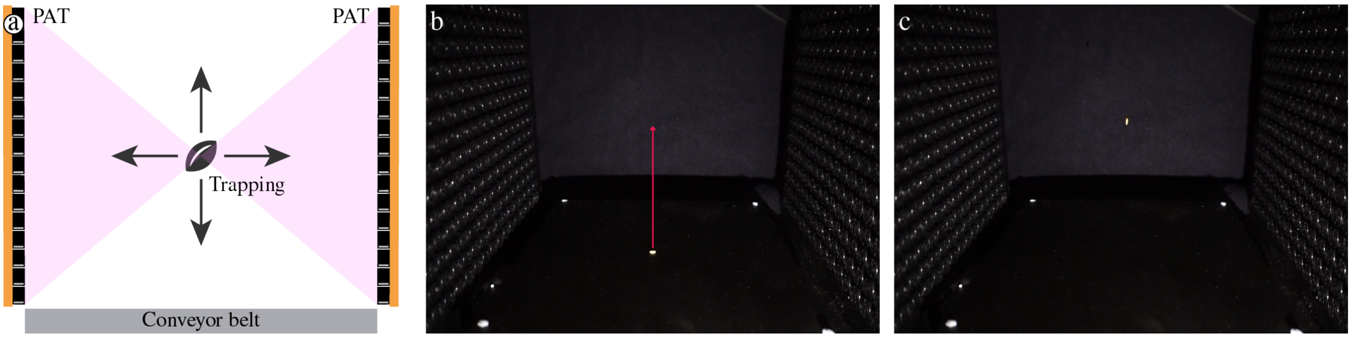

Figure 4: Preliminary results of the seed lifting application. **(a)** Setup overview. Note that the belt is moving in the direction of the figure's depth. **(b)** Single pepper seed is positioned on the surface. **(c)** Seed lifted by the PAT boards on the sides.

Supplementary Note 7. EFFECT OF PAT SIZE ON GENERATED FOCAL POINTS

We performed a simulated experiment with PAT boards of various size in which we focus the sound waves at (0, 0, 120 mm). We steadily decreased the number of transducers making up the board from 16×16 to 2×2 in steps of 2×2. All other parameters which matter and may affect the performance of the PAT (e.g., transducer spacing = 10.5 mm, diameter = 10 mm, frequency = 40 kHz) are all kept constant. The results of this experiment are displayed in Supplementary Fig. 5.

The PAT with 16×16 (256) transducers can create a very clearly defined focal point with a peak pressure around 7.5 kPa. As the number of transducers is reduced, the definition of the focal points as well as the peak pressure both steadily decrease. We show the xy pressure map of a focal point generated with a 16×16 transducer PAT board (b), the FWHM of that focus (c), relationship between number of transducers and peak pressure (d) and FWHM (e).

We measure FWHM by taking a cutline along the $y = 0$ line of an xy pressure plot (white dashed line in Supplementary Fig. 5b) at the focal plane (i.e. z=120mm). The peak pressure for the 16×16 case is ~ 7.5 kPa. The FWHM is then taken as the length of the red dashed line enclosed by the pair of green lines in Supplementary Fig. 5c. This process is repeated for each transducer arrangement and the results plotted in Supplementary Fig. 5d and Supplementary Fig. 5e. As we can see, the peak pressure shows a near-linear decreasing relationship along

with number of transducers. This makes sense as the pressure at a focal point is made up of contributions from each of the individual transducers in the board. The FWHM relationship is slightly more complex. At first, as transducer number decreases, the FWHM steadily increases in a near-linear fashion. This is because as the number of transducers drops, a less and less meaningful focus is able to be generated, and more dispersed beam shape is observed. At 6×6 and below, the board completely fails to be able to create a focus and FWHM ceases to be a meaningful measurement.

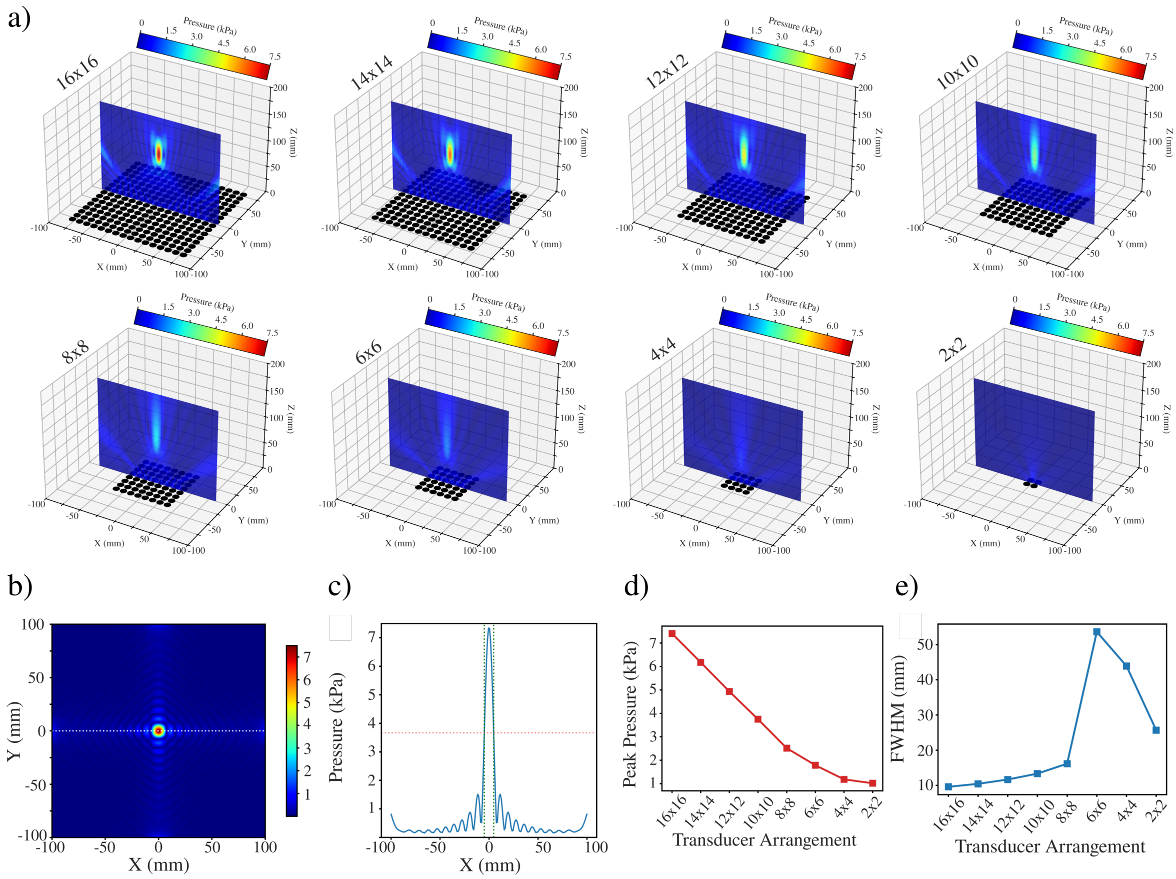

Figure 5: **(a)** Simulations of PAT boards focussing their acoustic pressure output at (0, 0, 12cm). The PATs are made up of steadily decreasing numbers of transducers. **(b)** XY slice of the focussed pressure coming from a PAT with 16×16 transducers. Taking a cutline of the pressure distribution at Y=0 results in **(c)**. The FWHM of this Gaussian-like distribution provides a measure for the size of the focal point. **(d)** Peak pressure of the focal points generated with each transducer arrangement. **(e)** FWHM of the focal points generated with each transducer arrangement. Source data for this figure are provided in Supplementary Data 4.

REFERENCES

[1] G. J. Laurent, A. Delettre, R. Zeggari, R. Yahiaoui, J. F. Manceau, and N. L. Fort-Piat, Micropositioning and fast transport using a contactless micro-conveyor, Micromachines, vol. 5, no. 1, 2014, DOI : 10.3390/mi5010066.

[2] R. Hirayama, D. Martinez Plasencia, N. Masuda, and S. Subramanian, A volumetric display for visual, tactile and audio presentation using acoustic trapping, Nature, vol. 575, no. January, 2019, DOI: 10.1038/s41586-019-1739-5.

[3] R. Hirayama, G. Christopoulos, D. Martinez Plasencia, and S. Subramanian, High-speed acoustic holography with arbitrary scattering objects, Science Advances, vol. 8, 2023, DOI: 10.1126/sciadv.abn7614.

[4] A. Marzo and B. W. Drinkwater, Holographic acoustic tweezers, Proceedings of the National Academy of Sciences of the United States of America, vol. 116, no. 1, pp. 84–89, 2019, DOI: 10.1073/pnas.1813047115.

[5] H. Bruus, Acoustofluidics 7: The acoustic radiation force on small particles, Lab on a Chip, 2012, DOI: 10.1039/c2lc21068a.
