# Supplementary material for: Acoustophoretic system for seed separation on conveyor belts
Source: Nat Commun. 2025 Jul 29;16:6975. doi: 10.1038/s41467-025-62006-3 (PMC12307790; doi:10.1038/s41467-025-62006-3)
Supplement: Supplementary file 2 — Description of Additional Supplementary Files [file 41467_2025_62006_MOESM2_ESM.pdf]

Description of Additional Supplementary File

Supplementary Data 1: Source Data for relevant panels in Figure 2.

Supplementary Data 2: Source Data for relevant panels in Figure 4.

Supplementary Data 3: Source Data for relevant panels in Figure 8.

Supplementary Data 4: Source Data for relevant panels in Supplementary Figure 5.

Supplementary Movie 1: A visual summary of our ultrasound-based seed-sorting system, showcasing the technology and its impact on agricultural automation.

Supplementary Movie 2: Initial results for pre-arrangement of seeds on a conveyor belt surface using acoustic hovering.

Supplementary Movie 3: Initial results showing acoustic levitation used to lift seeds from a conveyor surface into the air for inspection, coating, or priming.
